# Bisdemethoxycurcumin Induces Apoptosis in Activated Hepatic Stellate Cells via Cannabinoid Receptor 2

**DOI:** 10.3390/molecules20011277

**Published:** 2015-01-14

**Authors:** Phil Jun Lee, Seung Je Woo, Jun-Goo Jee, Sang Hyun Sung, Hong Pyo Kim

**Affiliations:** 1 School of Pharmacy, Ajou University, Suwon 443-749, Korea; E-Mails: phil@ajou.ac.kr (P.J.L.); zeus-03@hanmail.net (S.J.W.); 2 College of Pharmacy, Kyungbuk National University, Daegu 702-701, Korea; E-Mail: jjee@knu.ac.kr; 3 College of Pharmacy, Seoul National University, Seoul 151-742, Korea; E-Mail: shsung@snu.ac.kr

**Keywords:** bisdemethoxycurcumin (BDMC), curcumin, hepatic stellate cells (HSCs), liver fibrosis, cannabinoid receptor (CBR) 2, death-inducing signaling complex (DISC), adenosine triphosphate (ATP)

## Abstract

Activated Hepatic Stellate Cells (HSCs), major fibrogenic cells in the liver, undergo apoptosis when liver injuries cease, which may contribute to the resolution of fibrosis. Bisdemethoxycurcumin (BDMC) is a natural derivative of curcumin with anti-inflammatory and anti-cancer activities. The therapeutic potential of BDMC in hepatic fibrosis has not been studied thus far in the context of the apoptosis in activated HSCs. In the current study, we compared the activities of BDMC and curcumin in the HSC-T6 cell line and demonstrated that BDMC relatively induced a potent apoptosis. BDMC-induced apoptosis was mediated by a combinatory inhibition of cytoprotective proteins, such as Bcl_2_ and heme oxygenase-1 and increased generation of reactive oxygen species. Intriguingly, BDMC-induced apoptosis was reversed with co-treatment of sr144528, a cannabinoid receptor (CBR) 2 antagonist, which was confirmed with genetic downregulation of the receptor using siCBR2. Additionally, incubation with BDMC increased the formation of death-induced signaling complex in HSC-T6 cells. Treatment with BDMC significantly diminished total intracellular ATP levels and upregulated ATP inhibitory factor-1. Collectively, the results demonstrate that BDMC induces apoptosis in activated HSCs, but not in hepatocytes, by impairing cellular energetics and causing a downregulation of cytoprotective proteins, likely through a mechanism that involves CBR2.

## 1. Introduction

Medicinal plants have long been recognized as important sources of pharmaceutical drugs. As the demand for bioactive materials isolated from natural resources increases, a large amount of effort has been invested in screening phytochemicals with potential for treating human diseases such as liver fibrosis. Activation of hepatic stellate cells (HSCs) is a key event in the pathogenesis of liver fibrosis and leads to the accumulation of excess extracellular matrix. Excess deposition of extracellular matrix is directly linked to cirrhosis, liver failure and portal hypertension [1,2], often requiring liver transplantation. In the past, liver fibrosis was considered to be an irreversible process. Recently, cell death in activated HSCs was proposed as a potential approach that could be used to resolve liver fibrosis. 

One report evaluating the pathogenesis of liver fibrosis has suggested that cannabinoid receptors (CBRs) may play an important role in chronic liver disease [3]. A recent investigation showed that cannabidiol such as anandamide (AEA), naturally occurring compounds found in the *Cannabis sativa* plant, induces cell death of activated HSCs by activating CBRs [4,5]. These receptors are known to exist as two subtypes, named CBR1 and CBR2 [6]. The applicability of cannabinoids for treating patients with liver fibrosis is limited by their non-specific side effects, including the proposed increased risk of developing psychiatric disorders such as schizophrenia, depersonalization disorder and major depression [7]. 

Curcumin was recently reported to be capable of modulating the expression of CBR1 in hepatic tissue [8], suggesting a possible link between curcuminoids and cannabinoids. Curcumin isolated from the rhizome of *Curcuma longa* (turmeric) is widely used as a medicinal plant in China, India and other Asian countries [9]. Extensive studies have been carried out to explore the anti-oxidant, anti-inflammatory and anti-cancer properties of curcumin [10]. Interestingly, a number of studies have demonstrated that curcumin inhibits the proliferation of HSCs and induces their apoptosis, suggesting that curcumin may be a promising approach for treating liver fibrosis [11]. Interestingly, several studies have reported that cannabidiol, a CBR ligand, upregulates the levels of pro-apoptotic proteins, caspase-8, caspase-3, Bax and Bid in HSCs [12]. Curcumin is known to increase CBR1 expression, which can induce cell death of activated HSCs at higher doses. Both CBR1 and CBR2 are expressed in the liver. Notably, CBR2 is highly upregulated in the cirrhotic liver, predominantly in hepatic fibrogenic cells [13], implying that targeting CBR2 could be a promising strategy for the design of novel anti-fibrotic agents. However, the poor bioavailability and extensive metabolism of curcumin limits its therapeutic applications. A number of studies have demonstrated the anti-cancer effects of bisdemethoxycurcumin (BDMC), a naturally occurring demethoxy derivative of curcumin, in cells such as MCF-7 human breast cancer cells [14]. We therefore evaluated whether general curcuminoids, BDMC in particular, can induce cell death through CBR activation. The further experiments were conducted to compare the biological effects of BDMC with those of curcumin for apoptosis of activated HSCs. We observed that BDMC stimulates apoptosis in immortalized rat HSC-T6 cells by acting on CBR2. The apoptotic mechanism activated by BDMC likely involves CBR2-dependent formation of DISC. Additionally, decreasing intracellular ATP levels contributed to the induction of cell death. BDMC could therefore be a promising candidate for treatments that would resolve liver fibrosis. 

## 2. Results and Discussion

### 2.1. Results

#### 2.1.1. BDMC, but not Curcumin, Induces Apoptosis in Activated HSCs

**Figure 1:**
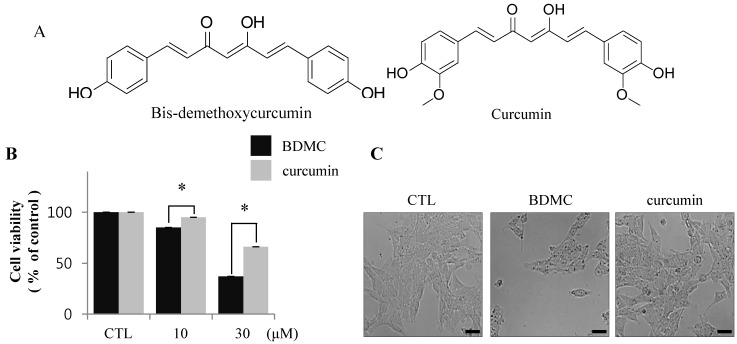

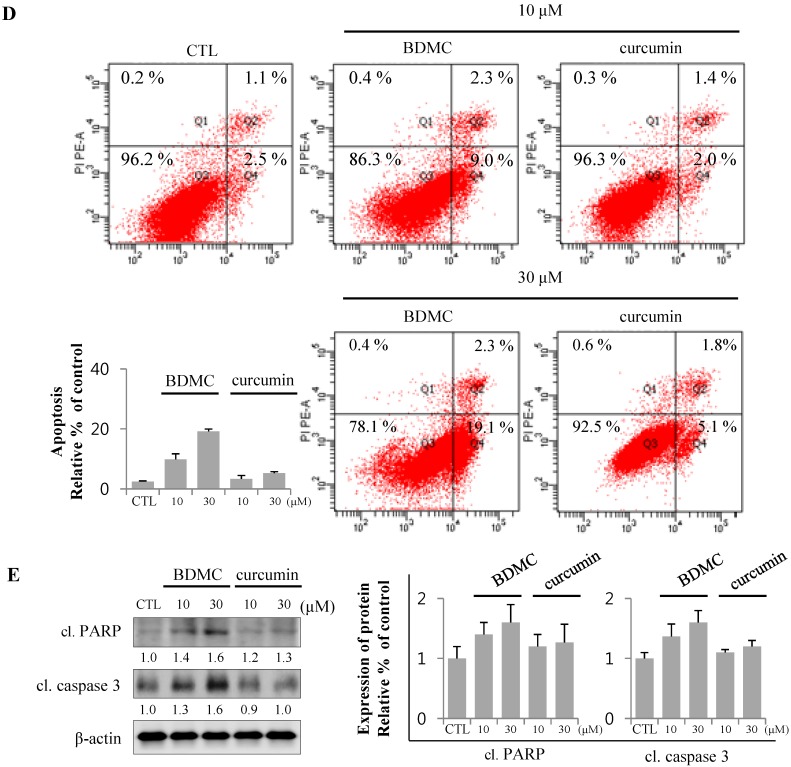
BDMC induces apoptosis in HSC-T6 cells. (**A**) The chemical structures of BDMC and curcumin; (**B**) Following treatment of cells with BDMC and curcumin (10 and 30 μM) for 24 h, MTT assay was performed to assess cell viability; (**C**) The change of morphology in cells treated with BDMC (30 μM) for 24 h was observed by light microscopy; (**D**) Cells were incubated with 10 and 30 μM of BDC or curcumin for 24 h and subsequently stained with Annexin-V/PI. The lower right quadrant (Annexin-V+/PI-) and upper right quadrant (Annexin-V+/PI+) present the percentage of cells in early and late apoptosis, respectively; (**E**) The expression of cleaved PARP and cleaved casapase-3 was analyzed by western blot, with β-actin used as a loading control. Data are expressed as mean ± S.D. (*n* = 3), * *p <* 0.05.

The structural difference between BDMC and curcumin is the presence of a dimethoxyl group at the *ortho*-position of the phenol rings of curcumin, as shown in Figure 1A. To evaluate the cytotoxic effects of BDMC and curcumin on activated HSCs, we examined the pro-apoptotic effect of BDMC on HSC-T6 cells, reflected as altered cell integrity, using MTT assay. HSC-T6 cells were exposed to BDMC or curcumin, each at 10 and 30 μΜ concentrations, for 24 h. We observed that BDMC, unlike curcumin, potently induced apoptosis in HSC-T6 cells (Figure 1B). After incubation with both compounds at 30 μΜ for 24 h, BDMC was found to significantly reduce the number of HSC-T6 cells (Figure 1C).

Flow cytometric analysis of damaged cells probed with Annexin-V/propidium iodide (PI) demonstrated that the cells underwent apoptosis following incubation with BDMC (Figure 1D). Conversely, induction of apoptosis in activated HSCs by curcumin was negligible at the low dose (10 μM; Figure 1C,D), but moderate at 30 μM. These results were confirmed by immunoreactivity staining for cleaved PARP and cleaved caspase 3, markers of apoptotic cell death (Figure 1E). Taken together, our findings show that induction of apoptosis in HSC-T6 cells was more prominent following incubation with BDMC than with curcumin. 

#### 2.1.2. Inhibition of Expression of Cytoprotective Proteins HO-1, Bcl_2_, Bcl-x_L_ and CBR2 by BDMC Contribute to Apoptotic Cell Death 

**Figure 2:**
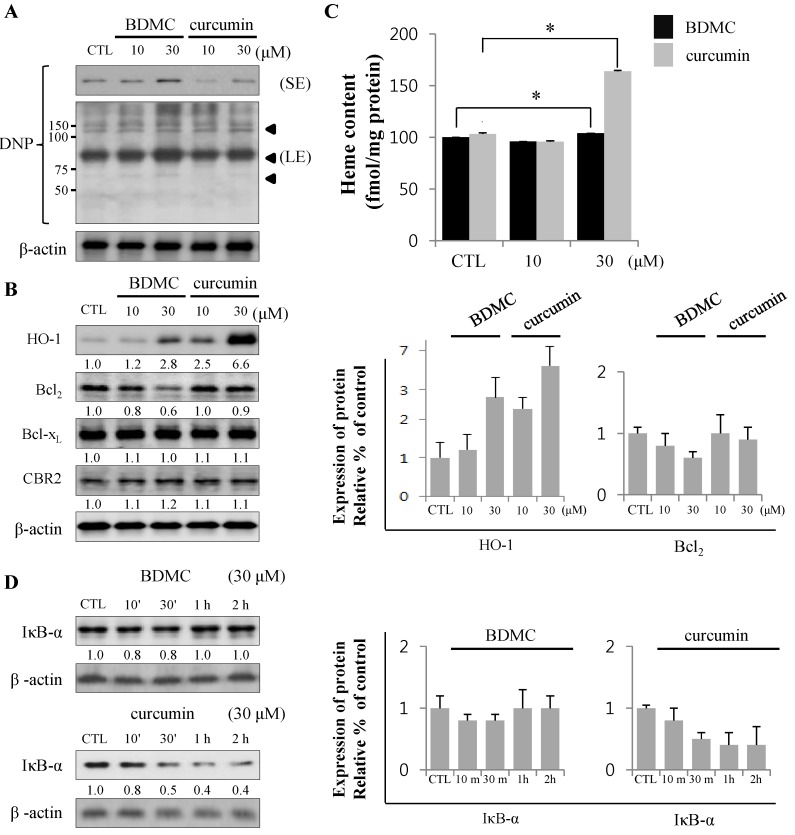
Expression of proteins regulating cell survival mediates apoptosis. (**A**) Relative production of reactive oxygen species (ROS) is shown using anti-2,4-dinitrophenol (DNP) with short and long exposure (SE and LE, respectively). B Black arrowheads indicate the increased ROS-binding unknown proteins; (**B**) The expression of HO-1, Bcl_2,_ Bcl-x_L_ and CBR2 was analyzed by Western blot; (**C**) Intracellular heme levels were measured by Heme colorimetric assay kit; (**D**) The expression of IκB-α was analyzed by Western blot, with β-actin used as a loading control. Data are expressed as mean ± S.D. (*n* = 3), * *p <* 0.05.

Since curcumin was shown to be the strongest inducer for Heme oxygenase (HO)-1 in several previous *in vitro* and *in vivo* experiments [15], we evaluated the effect of curcuminoids on the expression of this cytoprotective protein. As shown in Figure 2A, protein carbonylation is a type of oxidative modification of proteins promoted by reactive oxygen species (ROS).

**Figure 3:**
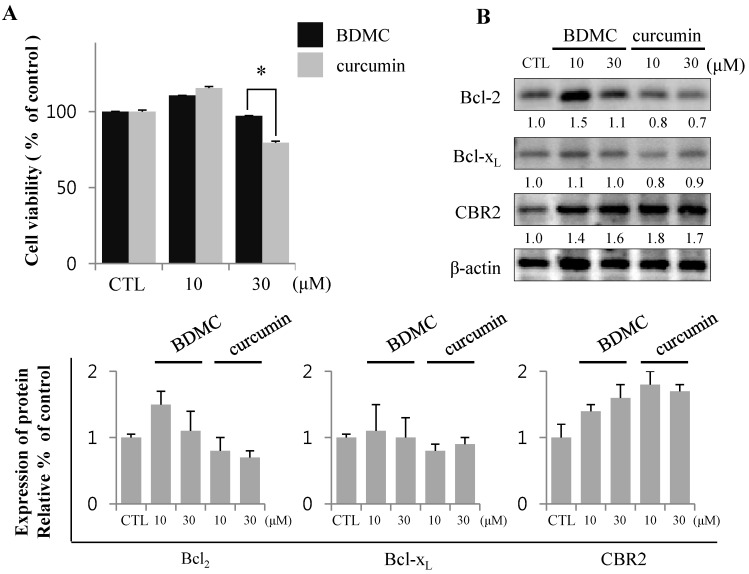
BDMC induces apoptosis in HSCs, but not HepG2. (**A**) HepG2 was treated with 10 and 30 µM of BDMC or curcumin for 24 h. MTT assay was performed to assess cell viability; (**B**) Intracellular levels of Bcl_2_, Bcl-x_L_ and CBR2 were measured by western blot with β-actin used as a loading control. Data are expressed as mean ± S.D. (*n* = 3), * *p* < 0.05.

Total lysates of HSC-T6 cells treated with curcuminoids were probed with anti-DNP antibody to assess the levels of oxidative stress in cells. Incubation with BDMC increased oxidative protein modifications (arrowheads in longer exposure blot) in HSC-T6 cells (Figure 2A), which were reflected in the apoptotic cell death (Figure 2B,D). The expression of HO-1 was robust in cells treated with curcumin. However, the induction of HO-1 by BDMC was moderate, even at higher doses. In contrast, the expression of anti-apoptotic Bcl_2_ protein was reduced in cells treated with BDMC (Figure 2B). Expression of Bcl-x_L_, an anti-apoptotic protein, was not altered by incubation with either curcuminoid. Additionally, CBR2 expression in HSC-T6 cells was not affected by treatment with curcuminoids. While intracellular heme levels were notably increased in curcumin-treated cells, the levels measured in BDMC-treated cells were almost equal to those in control cells (Figure 2C). Curcumin reduced the expression of inhibitor of NF-κB (IκB)-α protein, while BDMC treatment did not elicit an effect (Figure 2D). Reciprocal regulation of expression of cytoprotective proteins HO-1 and Bcl_2_ by curcuminoids and subsequent generation of ROS may preferentially trigger apoptosis in HSC-T6 cells. Curcumin was reported to suppress cell proliferation and induce apoptosis in human hepatoma cells (HepG2) [16]. To differentiate the effect of BDMC on selective cell death in HSCs from its effect on hepatocytes, we examined the cytotoxicity of curcuminoids in HepG2 cells. High dose (30 μM) of curcumin successfully induced cell death, as expected (Figure 3A). 

Conversely, BDMC did not affect HepG2 viability, but increased the expression of anti-apoptotic proteins (Figure 3B). These observations suggest that BDMC can induce apoptosis in HSCs, but not in the hepatocytes.

#### 2.1.3. Targeting of CBR2 by BDMC Mediates Apoptosis in HSC-T6 Cells

**Figure 4:**
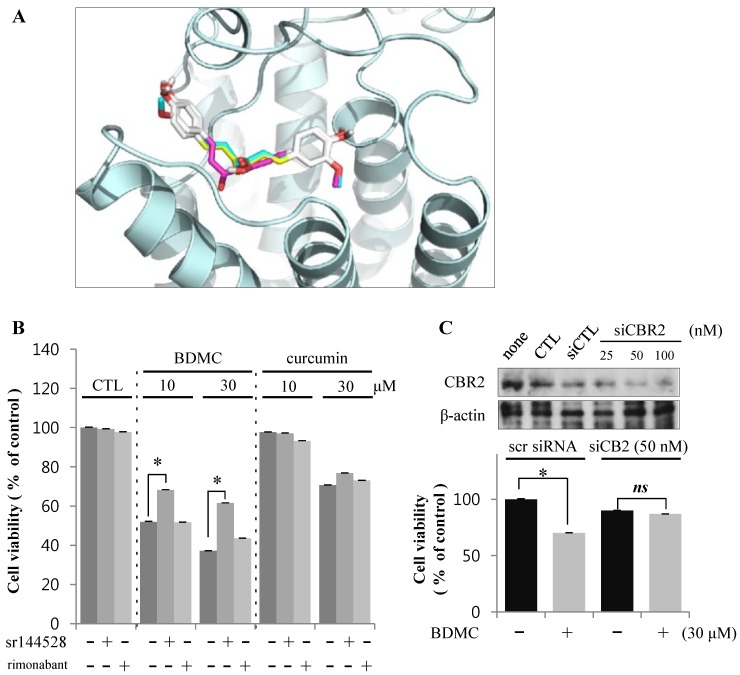
BDMC induces apoptosis of HSCs through an effect on CBR2. (**A**) Structure-based approach evaluating binding between curcuminoids and CBR2 using *in silico* modeling; (**B**) Cells were pre-incubated with CBR2 antagonist sr144528 (1 μM) for 2 h prior to treatment with 10 and 30 µM of BDMC or curcumin for 24 h; (**C**) Cells were transfected with CBR2 siRNA or control siRNA for 24 h. Data are expressed as mean ± S.D., * *p* < 0.05.

Curcumin has recently been demonstrated to affect hepatic expression of CBRs. The expression of both CBR1 and CBR2 isoforms was reported in HSCs [5]. Additionally, we tested the possible interaction of curcuminoids with CBR2. Using *in silico* analysis, we found that curcuminoids can bind CBR, suggesting that hydroxyl groups at both ends of the curcuminoid molecule may bind amino acid residues located at the active site of CBRs (Figure 4A). 

To identify the specific CBR isotypes involved in the cytotoxicity of BDMC, we treated HSC-T6 cells with curcuminoids in the presence or absence of CBR antagonists. Interestingly, only sr144528, a CBR2 antagonist, reversed the apoptotic activity of BDMC (Figure 4B). Rimonabant, a CBR1 antagonist, elicited no detectable effect on cell death in HSC-T6 cells. Genetic downregulation of CBR2 by small interference (si) RNA also ameliorated the viability of HSC-T6 cells following BDMC treatment (Figure 4C). On the basis of these observations, we propose that BDMC-induced apoptosis of activated HSCs may be mediated via CBR2.

#### 2.1.4. BDMC-Induced DISC Formation in HSCs

**Figure 5:**
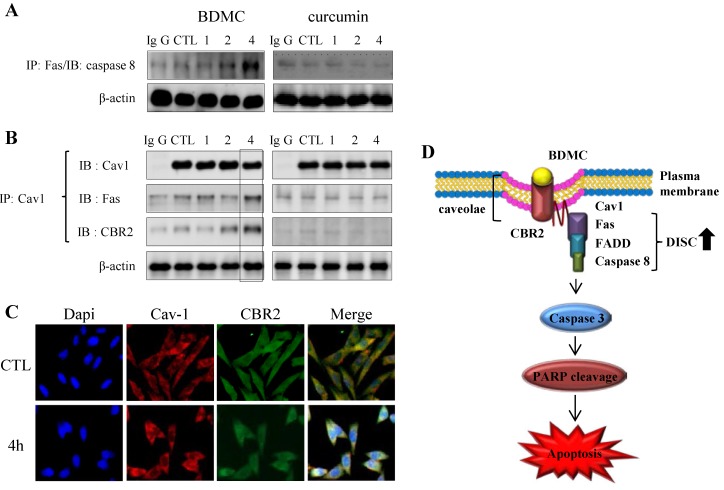
The CBR2-dependent formation of cav-1 with DISC complexes. (**A**) Cells were subjected to immunoprecipitation with anti-Fas prior to detection of anti-caspase-8; (**B**) or with cav-1 prior to detection of CBR2 and Fas; (**C**) Trafficking of cav-1 and CBR2 following treatment with BDMC was evaluated using confocal microscopy; (**D**) A schematic representation of the formation of the CBR2-dependent DISC. β-actin was used as a loading control.

The contribution of the extrinsic apoptotic pathway to BDMC-induced cell death in HSC-T6 cells has been investigated. Fas triggers Fas-dependent death pathway, characterized by the formation of death-inducing signaling complex (DISC). The recruitment of caspase-8 to the DISC, followed by its activation, leads to a subsequent activation of effector caspases, which in turn results in cell death [17]. BDMC acutely augmented the formation of DISC in HSC-T6 cells (Figure 5A). 

In line with this observation, interaction of cav-1 with both Fas and CBR2 was observed only in BDMC-treated HSC-T6 cells (Figure 5B). Caveolin-1 (cav-1) is an integral membrane protein and the main structural protein of caveolae in various cells [18]. Based on the fact that CBR2 contains the typical cav-1-binding motif [19], it was suggested that cav-1 may be involved in CBR2-dependent apoptosis. BDMC treatment appeared to increase the colocalization of CBR2 with cav-1, which was further upregulated at 4 h (Figure 5C). Under the same conditions, we did not observe any interactions between cav-1 and Fas or CBR2 in curcumin-treated cells (Figure 5A,B). CBR1 was reported to colocalize with cav-1 in neuronal cells [20].

#### 2.1.5. BDMC Reduces Cellular ATP Levels by Depleting ATP

**Figure 6:**
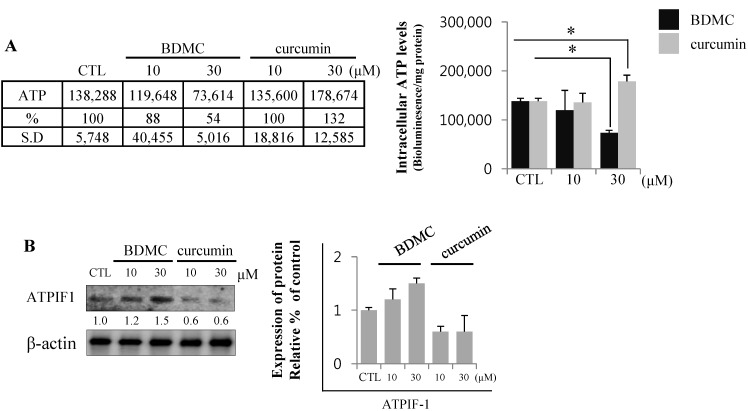
BDMC treatment decreased ATP levels by blocking ATP synthase. (**A**) BDMC treatment impaired cellular energetics in HSC-T6 cells; (**B**) The expression of ATPIF1 was evaluated by western blot; using β-actin as a loading control. Data are expressed as mean ± S.D., * *p* < 0.05.

Mitochondrial membrane potential (MMP) is the decisive factor that determines cellular survival or death. MMP is maintained by a proton gradient generated across a mitochondrial inner membrane, which drives ATP production. Oxidative stress inhibits MMP formation, which induces cell death by depleting intracellular ATP levels [21]. Cellular ATP levels are therefore an important parameter in the initiation and progression of cell death [22]. The endogenous levels of ATP reflect the presence of cell injury, with BDMC significantly reducing ATP levels in HSC-T6 cells. Cellular ATP was depleted to approximately 50% of the baseline levels upon treatment with 30 μM of BDMC (Figure 6A). Moreover, the expression of ATPase inhibitory factor (ATPIF) 1 increased following BDMC treatment, possibly accounting for the dose-dependent decrease in cellular ATP levels. Conversely, the effect of curcumin on ATPIF1 protein expression was found to be negligible (Figure 6B). 

### 2.2. Discussion

Our current study demonstrates that BDMC induces apoptosis in HSC-T6 cells through an action involving CBR2. BDMC, a curcumin-derivative, is characterized by the lack of methoxyl groups at the *ortho* position on the aromatic ring. We have shown BDMC to be a more potent inducer of apoptosis in HSC-T6 cells. Furthermore, we have demonstrated that BDMC-induced apoptosis involves the regulation of Bcl_2_ expression, ROS production and CBR2-dependent formation of DISC. Therefore, we propose that BDMC treatment could be useful in chronic liver diseases.

Chronic liver injuries reflect a continuous cycle of acute liver damage and wound healing, extended over time. Perpetuated cycles of injury and healing result in the emergence of a fibrotic phenotype of the liver, with progressive fibrotic changes previously considered to be a one-way process. With a recent paradigm shift in hepatic treatment towards the consideration of hepatic fibrosis as reversible, a combination of inhibition and careful control of injury has become an increasingly promising therapeutic strategy, aimed at preventing end-stage liver diseases [23]. In the normal liver, the major function of HSCs is the storage of vitamin A. However, HSCs undergo activation and trans-differentiation to myofibroblasts upon liver damage, leading to liver fibrosis [24]. Apoptosis of HSCs, but not hepatocytes, may contribute to the termination of fibrogenesis during the resolution of fibrosis [25]. Therefore, a number of attempts have been made to identify natural products that would be able to kill activated HSCs and thereby contribute to the resolution of liver fibrosis. Accumulating evidence suggests that curcumin, a naturally occurring chemical compound found in turmeric (*Curcuma longa* L.), induces apoptosis in activated HSCs. However, little is currently known about the possible effects of BDMC on apoptosis in activated HSCs.

We investigated whether BDMC and curcumin regulate the expression of intracellular proteins including HO-1, Bcl_2_ and Bcl-x_L_ that can modulate cell death. Prominent differences in the effects of tested compounds on Bcl_2_ and HO-1 expression in HSC-T6 was observed, with the expression of Bcl_2_ being more reduced and intracellular HO-1 levels less increased in BDMC-treated cells, compared to curcumin-treated cells. These findings suggest that BDMC could shift the balance toward apoptosis in activated HSCs. Interestingly, current study demonstrated that treatment with curcumin, but not BDMC, markedly increases intracellular heme levels. However, curcumin-induced increase in heme levels did not result in notable induction of cellular damage in activated HSCs. These findings suggest that upregulation of HO-1 levels by curcumin is sufficient to catalyze heme into cytoprotective by-products, namely bilirubin and carbon monoxide (CO) [26]. Upregulation of HO-1, an enzyme with widely recognized cytoprotective properties, plays a critical role in decreasing ROS levels and degrading cytotoxic heme into CO and iron [27]. Since heme is both an inducer and a substrate of HO-1 cytoprotective metabolites, it is difficult to distinguish which step occurs first, namely the removal of cytotoxic heme or the induction of the cytoprotective enzyme.

Our study demonstrates that BDMC-induced apoptosis in HSC-T6 cells was reversed by a co-treatment with a CBR2 antagonist, sr144528, but not with a CBR1 antagonist. Similarly, BDMC-induced apoptosis was prevented by genetic downregulation of the receptor using siCBR2.

AEA acts as an endogenous agonist for CBR1 and CBR2 to induce apoptosis in HSCs, but not in hepatocytes [12]. To evaluate whether CBR2-specific action of BDMC induces apoptosis only in HSC-T6 cells, we performed an experiment evaluating the effect of BDMC on HepG2 cells, which are recognized as a suitable *in vitro* model system for the study of polarized human hepatocytes [28]. Cell viability following treatment with BDMC was almost equal to that observed in untreated control cells. However, curcumin induced cell death of HepG2 in a dose-dependent manner. These results suggest that BDMC may be a candidate agent for developing pharmaceuticals with an aim of inducing selective apoptosis of activated HSCs by targeting CBR2.

Additionally, we investigated the mechanism by which CBR2-targeting compounds induce cellular damage in activated HSCs. Our study demonstrated that treatment with BDMC causes the formation of DISC and a decline in intracellular ATP levels by increasing ATPIF1 expression. ATPIF1 inhibits ATPase by switching cellular metabolism to ATP hydrolysis during the collapse of the membrane proton electrochemical gradients in the mitochondria [29]. At a fundamental level, apoptosis is regulated through two pathways [30]. The extrinsic pathway is initiated by the activation of a death receptor, leading to the recruitment of FADD and procaspase-8 to form DISC. DISC formation leads to autocleavage and subsequent activation of caspase-8, which, in turn, activates caspase-3 or caspase-7, leading to apoptosis. The intrinsic pathway is characterized by mitochondrial damage resulting in the release of cytochrome c and subsequent formation of an apoptosome composed of apoptosis protease activator factor-1, dATP and procaspase-9. Apoptosome formation is followed by a serial activation of caspase-3, -7 and -9, eventually leading to apoptosis. BDMC-induced apoptosis of HSC-T6 cells was accompanied by DISC formation and a decrease in ATP levels, which may reflect a possible cross-talk between the two pathways of apoptosis. Apoptotic events that are regulated by the effect of BDMC on CBR2 signaling in activated HSCs appear to involve the formation of DISC. 

The colocalization and interaction of cav-1 and Fas were reported during hypoxia-induced apoptosis in Beas-2B cells, promoting the formation of DISC [31]. We recognized the existence of cav-1-binding motif in the amino acid sequence of CBR2 (206YTYGHVLW215), but not CBR1. AEA is known to be internalized and recycled by caveolin-rich membrane domains [32]. Our study shows that both CBR2 and Fas can bind to cav-1. However, CBR2 is not likely to directly bind to Fas (Figure 5B). While neither BDMC nor curcumin were found to alter cav-1 expression, BDMC increased co-localization of cav-1 with CBR2 and Fas respectively. Taken together, our findings suggest that cav-1 may play an essential role in CBR2-mediated BDMC-induced apoptosis.

## 3. Experimental Section

### 3.1. Chemicals and Reagents

An immortalized rat hepatic stellate cell (HSC) line HSC-T6 was kindly provided by S.L. Friedman (Columbia University, New York, NY, USA). The immortalized HSC-T6 cell line has been known to retain virtually all features of activated HSCs.

Human liver carcinoma cell line (HepG2) was supplied by Korea Cell Line Bank (KCLB, Seoul, Korea). HSCs and HepG2 were cultured according to KCLB directions in Dulbecco’s modified Eagle’s medium (DMEM) with 10% Fetal Bovine Serum (FBS) and 1% Penicillin Streptomycin (Pen Strep) in a humidified atmosphere of 5% CO_2_/balanced air at 37 °C. Curcumin was isolated from the rhizome of Curcuma longa Linn. Bisdemethoxycurcumin was purchased from Cayman Chemical (Ann Arbor, MI, USA).

### 3.2. MTT Assay

The effects of curcuminoids on the viability of activated HSCs were determined by the 3-(4,5-dimethylthizaol-2-yl)-2,5-diphenyltetrazolium bromide (MTT) assay. In each experiment, cells were plated in 100-µL aliquots of growth medium into 96-well plates at 10^5^ cells per well and incubated for 24 h. Curcuminoids were added to each well at a concentration of 0, 10, or 30 μΜ). MTT solution (5 mg/mL) was then added to each well, the formazan precipitate was dissolved in 100 µL of dimethyl sulfoxide and the cells were incubated for 2 h. Following incubation, the absorbance was measured using an automated microplate reader (Bio-Tek, San Diego, CA, USA) at 562 nm. The relative cell survival (%) was calculated as a ratio between absorbance of treated and control (untreated) cells, expressed as a percentage. The experiments were performed at least 3 times, with each condition plated in triplicate.

### 3.3. Western Blot Analysis and Antibodies

Cells were lysed with radioimmunoprecipitation (RIPA) assay buffer containing 1× PBS, 1% (*v*/*v*) Nonidet P-40 (NP-40), 0.5% (*w*/*v*) sodium deoxycholate, 0.1% (*w*/*v*) sodium dodecyl sulfate (SDS), 0.1 mg/mL phenylmethylsulfonyl fluoride (PMSF), 30 μL/mL aprotinin and 1 mM sodium orthovanadate (Na2VO3). Cell lysates were centrifuged and the resulting supernatants collected. Proteins were separated on an 8%–15% SDS–polyacrylamide gel and transferred to a polyvinylidene difluoride (PVDF) membrane. Membrane was blocked in Tris-buffered saline (TBS) containing 0.1% Tween 20 (TBST) and 5% non-fat dry milk for 1 h at room temperature and then incubated overnight with primary antibodies in TBST containing 1% non-fat dry milk at 4 °C. Anti-cleaved caspase-3, anti-HO-1, anti- Bcl_2_, anti- Bcl-x_L_, anti-CBR2, anti-ATPIF1 and anti-β-actin were purchased from Santa Cruz Biotechnology (Santa Cruz, CA, USA). Anti-cleaved poly ADP-ribose polymerase-1 (PARP) antibody was purchased from Cell Signaling Technologies (Danvers, MA, USA). Anti-dinitrophenol was purchased from Abcam (Cambridge, MA, USA). Membranes were washed with TBST and incubated with goat anti-rabbit or anti-mouse horseradish peroxidase-conjugated IgG secondary antibody for 2 h. Signal was quantified using the chemiluminescence system (GE Healthcare, Piscataway, NJ, USA). Bands were quantified by densitometric analysis using Image J software. Data are representative of three independent experiments.

### 3.4. Apoptosis Analysis 

After treatment with BDMC or curcumin at 0 (control), 10 and 30 μM, cells were detached with ethylenediaminetetraacetic acid (EDTA)-free trypsin and washed twice with cooled PBS. Cells were re-suspended in 400 µL of 1× loading buffer with 5 µL of Annexin-V and 5 µL of propodium iodide (PI; Becton-Dickinson, San Diego, CA, USA) for 15 min on ice in the dark. Analyses were performed using FACS Calibur analyzer (Becton-Dickinson, San Diego, CA, USA).

### 3.5. Intracellular Heme Levels

Intracellular heme levels were measured using a heme colorimetric assay kit (BioVision, Mountain View, CA, USA). Briefly, cells were harvested, counted and lysed in lysis buffer. Aliquots (50 µL) from each diluted sample or standard were transferred into 96-well plates prior to the addition of 50 µL of heme assay kit solution to each well. After incubation for 30 min, the light emitted was measured at 570 nm using an automated microplate luminometer (Bio-Tek, San Diego, CA, USA). 

### 3.6. Immunoprecipitation

Cells were rinsed in PBS, harvested and lysed in lysis buffer containing 10% glycerol, 0.5 mM EDTA, 25 mM Tris-HCl (pH 8.0), 150 mM NaCl, 1 mM Na_2_VO_3_, 10 mM β-glycerophosphate, 0.1% NP-40 and 1 mM dithiothreitol in the presence of protease inhibitors and 1 mM PMSF. Cell extracts were incubated with the specific anti-CBR2 antibody overnight at 4 °C, followed by incubation with protein A/G agarose beads (Santa Cruz). Following incubation, cells were extensively washed and separated by electrophoresis. Proteins were transferred onto PVDF membranes and Western blotting was performed following standard protocols.

### 3.7. Confocal Microscopy

Activated HSCs were settled on a glass coverslip at a density of 2 × 10^4^ cells/cm^2^ and treated with 30 µM BDMC for 4 h. Cells were fixed for 10 min at room temperature with 4% paraformaldehyde and processed for immunofluorescence. Rabbit anti-CBR2 antibodies (diluted 1:100) and mouse anti-cav-1 antibodies (1:100) were made fluorescent using the Alexa Fluor 488 and 546 Monoclonal Antibody (Invitrogen, Carlsbad, CA, USA). Following immunofluorescence, coverslips were mounted using VECTASHIELD (Vector Laboratories, Inc., Burlingame, CA, USA) and data were acquired with a C1 confocal microscope (Nikon Instruments S.P.A., Florence, Italy) at excitation wavelengths of 488 (band of Ar laser) or 546 nm (band of a HeNe laser).

### 3.8. Measurement of Intracellular ATP Levels

Intracellular ATP levels were measured using the ATP Bioluminescence Assay kit HS II (Roche Applied Science, Monza, Italy). Briefly, cells were harvested, counted and lysed with lysis buffer. Aliquots (50 µL) from each diluted sample or standard were transferred into 96-well plates and 50 µL of luciferase reagent was added. After mixing, the light emitted was measured and integrated over 10 s using an automated microplate luminometer (Bio-Tek). 

### 3.9. Statistical Analysis

All values are expressed as means ± standard error. Statistical significance was determined by the Student’s t-test, with *p* < 0.05 considered significant.

## 4. Conclusions

Taken together, our data indicate that BDMC promotes apoptosis in activated HSCs by activating a CBR2-dependent apoptotic pathway. In addition, CBR2 could be a novel molecular target of curcumininoid. Although further mechanistic studies are required to examine this anti-fibrotic effect, BDMC may have therapeutic potential for use in treatment of hepatic fibrosis.
